# A Study on Millimeter Wave SAR Imaging for Non-Destructive Testing of Rebar in Reinforced Concrete

**DOI:** 10.3390/s22208030

**Published:** 2022-10-20

**Authors:** The-Hien Pham, Kil-Hee Kim, Ic-Pyo Hong

**Affiliations:** 1 Department of Smart Information Technology Engineering, Kongju National University, Cheonan 31080, Korea; 2 Department of Green Smart Architect Engineering, Kongju National University, Cheonan 31080, Korea

**Keywords:** SAR imaging, non-destructive testing, compressed sensing, millimeter wave

## Abstract

In this study, we investigate a millimeter wave (mmWave) synthetic aperture radar (SAR) imaging scheme utilizing a low-cost frequency modulated continuous wave (FMCW) radar to take part in non-destructive testing which could be a useful tool for both civilian and military demands. The FMCW radar working in the frequency range from 76 GHz to 81 GHz is equipped with a 2-D moving platform aiming to reconstruct the 2-D image of the shape of the target object. Due to the lab environment containing several devices and furniture, various noise and interference signals from the floor are not avoidable. Therefore, the digital signal processing algorithms are joined to remove the undesired signals as well as improve the target recognition. This study adopts the range migration algorithms (RMAs) on the processed reflected signal data to form the image of the target because of its verified ability in this type of mission. On the other hand, the integration of compressed sensing (CS) algorithms into the SAR imaging system is also researched which helps to improve the performance of the system by reducing the measurement duration while still maintaining the image quality. Three minimization algorithms are used involving the imaging system as the CS solvers reconstruct the radar data before being processed by RMA to form the image. The proposed imaging scheme demonstrates its good ability with high azimuth resolution in the mission of detecting tiny cracks in the rebar of reinforced concrete. In addition, the participation of CS algorithms improves the performance of the scheme as the cracks on the rebar can be located on the images, which are reconstructed from only 30% of the dataset. The comparison of CS solvers shows that ADMM outperforms the other candidates in the reconstruction task.

## 1. Introduction

According to international regulations, to ensure the serviceability of any building materials, products, and elements over their entire useful lives, their specific physical and strength properties should be defined [1]. Hence, the constant monitoring of the structures such as lifetime assessment of industrial construction plays a crucial role in the guarantee of safety [2]. Although it has the disadvantage of being an indirect method, non-destructive testing (NDT) is widely applied in civil engineering because of its beneficial characteristics, including its non-destructive nature, repeatability, and economical aspect. NDT methods can conduct a condition assessment to provide information for the structural performance of the material, e.g., equipment integrity analysis, corrosion monitoring of structures and equipment, corrosion damage evaluation, fatigue and creep damage prediction, and fitness-for-service evaluations [2]. There are various types of NDT methods employed in routine services for the integrity of structural component detection, such as radiography testing, eddy current testing, ultrasonic, and resonant near-field probes. However, these methods not only require expertise but have their own limitations. For example, the operator safety factor must be taken into account as they must pay careful attention while using the radiography method [3]. On the other hand, eddy current is known as one of the effective NDT techniques for testing the steel members [4,5], which is based on the change of voltage or current in the sensing coil. Nevertheless, a highly skillful and experienced inspector is one of the main requirements. Additionally, some special signals occur as the defect at the root of threads and the signal lags severely as the increment of the detection depth [6,7] makes them hard to process. The ultrasonic method is based on measuring the transit time that is required for the ultrasonic pulse to go through the material several times. Hence, the knowledge about the precise physical characteristics of the material, such as the ultrasonic velocity, plays an important role [8] which can lead to inaccurate inspection results [9].

Recently, SAR imaging has become one of the most effective NDT methods and appealed to more and more attention not only in ground or sea detection but also short distance applications including indoor detection or structure health monitoring (SHM) since it takes advantage of the natural characteristics of the electromagnetic (EM) wave with good penetration performance. In general, radars are utilized to emit the EM wave for both civilian and military applications in the form of impulse radars and continuous wave (CW) radars. In terms of CW radars, the FMCW radar is preferable as this type of radar is not restricted to military applications as well as it can be adapted for commercial tasks such as measuring material thickness [10], monitoring terrain displacement [11,12], see-through-wall applications [13], life activity monitoring [14], as well as automotive applications in collision avoidance [15], controlling adaptive cruise [16], and all-weather cruise [17]. Moreover, FMCW radars are extensively employed in imaging applications. The combination of FMCW radar and SAR imaging techniques results in a low-cost, effective imaging sensor with high resolution. The research in [18] also studies the application of SAR imaging in detecting the rebar in the reinforced concrete but due to the low resolution of the imaging scheme, it can only identify the presence of the rebar without recognizing any defects. The authors in [19] proposed a 2-D array antenna system to reconstruct the SAR images of small structures, which may be a highly cost-effective method. In [20], a scheme employing FMCW SAR imaging for the NDT application to detect a defect on the 3-D printed object was presented. However, a huge number of measurements in the experiment can cause a time consumption problem.

The appearance of CS in the previous two decades [21,22] has brought many CS-based potential applications not only for image processing but also for signal processing, and SAR imaging is also one of the candidates [23,24]. The author in [25] proposed an intensity-hue-saturation-based (IHS-based) pan-sharpening method using ripplet transform and CS to solve the evident spectral distortion problem caused by the IHS transform. In this research, the CS is adopted to reconstruct the intensity component to integrate the local information from both the intensity component and the panchromatic (PAN) image. The research in [26] studies the integration of CS in spare reconstruction on the randomly sub-sampled raw data of automobile FMCW SAR for far-field imaging. Their CS scheme is based on the basis pursuit denoising (BPDN) solver to recover the Ku-band FMCW signal data. CS was also utilized by the study in [27] aiming to archive the high azimuth resolution for the low-THz band using the convex solver. By applying the CS method, the SAR image can be reconstructed even though the radar data are acquired in an irregular spatial interval. The CS-SAR imaging scheme can form the images of sparse or compressible targets from a randomly under-sampled data collection and thus it can help to reduce the number of measurements efficiently. The study in [28] applies the CS to recover the FMCW signals before SAR processing from two of the in-phase and quadrature (I/Q) components using the $l_{1}$-norm minimization [29], which may take a considerable duration of the recovery process. In this study, we adopted the RMA algorithm as the SAR imaging construction technique which has been proven as an effective approach in near-field SAR imaging [30] to identify the location of a crack in the steel rebar which is used in reinforced concrete. The 2-D shape of the object is formed in the image result, which causes numerous measurements to accumulate adequate signal data. Therefore, by directly applying CS to the complex signal data gathering from the measurement platform, this study investigates a method to reduce the great number of measurements and the side-lobe effect caused by the non-uniform spacing of measurement points [31], which result in saving the measurement and the processing period while maintaining the quality of the images. Additionally, we investigated three different CS-based solvers, namely the alternating direction method of multipliers (ADMM), iterative reweighted least squares (IRLS), and coordinate descent (CD) to recover the signal data from small random measurement collections. To maintain the image quality and reduce environmental interference, the finite impulse response (FIR) filter along with the smoothing window and signal-to-noise improvement techniques are employed. The quality of the images formed from the data recovered by the CS-based methods was compared to that of the original image which was reconstructed by utilizing the entire data. In addition, the effectiveness of different CS-based algorithms is also revealed.

This paper is organized as follows: in Section 2, the methodology of the proposed imaging scheme is presented. Section 3 describes the experiment setup along with the results. Finally, Section 4 gives the summarization and conclusion of this paper as well as the future works to enhance the system performance.

## 2. Theory and Methodology

**Figure 1:**
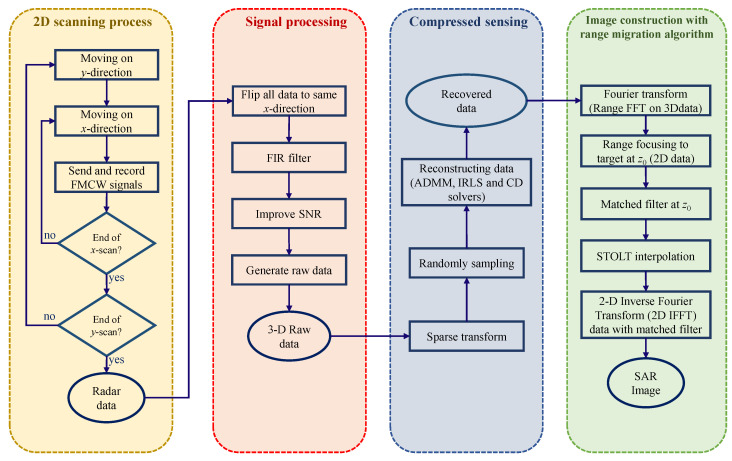
Block diagram of the imaging system.

In this section, we describe the theoretical approaches which are employed in our study. We first present the FMCW echo containing the reflectivity information of the target scatterer. Then, the method of adopting the signal data to reconstruct the SAR image by RMA is depicted followed by the compressed sensing algorithms to improve the performance of our imaging scheme. The overall process of the proposed scheme is illustrated in Figure 1.

### 2.1. FMCW Signal and RMA Approach

**Figure 2:**
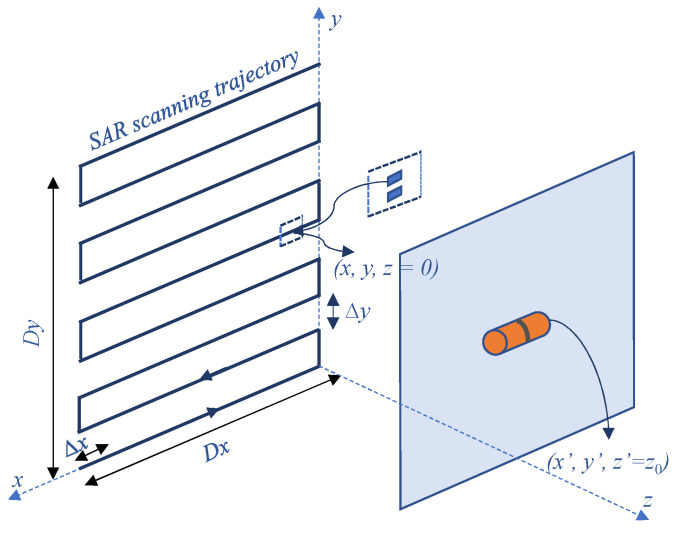
Target measurement using mmWave radar system.

The characteristics of the FMCW signal that can be utilized by SAR imaging are presented in this section. As shown in Figure 2, an (x,y,z) Cartesian coordinate system is established consisting of the *x*-axis, *y*-axis, and *z*-axis, which denote horizontal, vertical, and range directions, respectively. We assume that a single scatterer located at position $\left(x^{\prime },y^{\prime },z_{0}\right)$ with a complex reflectivity $\rho (x^{\prime },y^{\prime })$ while the location of the FMCW radar is $\left(x_{R},y_{R},0\right)$. The radar sensor moving on the *Oxy* plane results in the rectangle grid of measurement points. The radar is considered to carry a single full-duplex antenna at the mid-point between the transmitting and receiving antennas since they are so close to each other [32]. In the FMCW SAR imaging configuration, by performing the data collection at all measurement points, a 3-D (three-dimensional) data cube can be accumulated as the backscattered signal data. The intermediate frequency (IF) signal at each measurement point is denoted as:

(1) $$\begin{array}{c} s_{IF}\left(x,y,t\right)=\frac{\rho (x^{\prime },y^{\prime })}{R^{2}}\exp j2\pi (f_{0}\tau +\alpha t\tau -\frac{1}{2}\alpha \tau^{2}) \end{array}$$

where *R* is the distance between the radar sensor and the scatterer, $f_{0}$ is the carrier frequency at 0, τ is the time delay between the transmitted and the received signal with the speed of light *c*, and α is the slope of the frequency in the chirp duration *T* with the bandwidth *B*. It is clearly seen that the IF signal here is a sinusoidal signal with the beat frequency $f_{b}=\alpha \tau$ which is proportional to the time delay (τ) so that it can provide the range information. The residual video phase (RVP), which is the last term in (1), is negligible and can be ignored [33]. Therefore, the IF signal can be redefined in the wavenumber domain as:

(2) $$\begin{array}{c} s_{IF}\left(x,y,k\right)=\rho \left(x^{\prime },y^{\prime }\right)\frac{\exp j2kR}{R^{2}},\;\frac{2\pi f_{0}}{c}\leq k\leq \frac{2\pi f_{T}}{c} \end{array}$$

where $f_{T}=f_{0}+\alpha T$ is the maximum swept frequency of FMCW radar and is the corresponding wave number at each specific frequency *f*.

As a 2-D target can be characterized by utilizing the reflectivity function $\rho (x^{\prime },y^{\prime })$, the RMA employs the $\rho (x^{\prime },y^{\prime })$ at all measurement points to form the image of the target object. Here we assume that the measurement system continuously captures data over the Oxy plane which covers the target dimension. Hence, the backscattered signal data from the target plane at $z_{0}$ distance can be derived by taking the integral of (2)

(3) $$\begin{array}{c} s\left(x,y,k\right)=\;\int \int \;\rho \left(x^{\prime },y^{\prime }\right)\frac{\exp(j2kR)}{R^{2}}dx^{\prime }dy^{\prime } \end{array}$$

where the distance between the transceiver antenna and the general point on the target at distance $z_{0}$ from the aperture plane is $R=\sqrt{{\left(x-x^{\prime }\right)}_{(}^{2}y-y^{\prime }{)}^{2}+z_{0}^{2}}$. As the considered single full-duplex antenna, $R^{2}$ in (3) is approximately $\left(z_{0}R^{-1}\right)$. Due to the near-field application adopted in this study, the error in this approximation can be negligible [34]. In addition, $z_{0}$ is the constant and as the target is stationary, it can also be ignored. As a result, the signal data for image construction will be processed by a combination of phase terms and the remaining $R^{-1}$ dependence. Now, the backscattered signal is

(4) $$\begin{array}{c} s\left(x,y,k\right)=\;\int \int \;\rho \left(x^{\prime },y^{\prime }\right)\frac{\exp(j2kR)}{R}dx^{\prime }dy^{\prime } \end{array}$$

According to Weyl’s study in [35], there is a representation of a spherical wave as the superposition of plane waves. In the case of the homogeneous plane waves, the backscattered signal can become

(5) $$\begin{array}{c} s\left(x,y,k\right)=\frac{j}{2\pi }\;\int \int \;℘\left(k_{x},k_{y}\right)\times \frac{\exp(jk_{z}z_{0})}{k_{z}}\exp\left(j\left(k_{x}x+k_{y}y\right)\right)dkxdky \end{array}$$

where

(6) $$\begin{array}{cc} ℘(k_{x},k_{y}) & =\;\int \int \;\rho \left(x^{\prime },y^{\prime }\right)\exp\left(-j\left(k_{x}x^{\prime }+k_{y}y^{\prime }\right)\right)dx^{\prime }dy^{\prime }\\ \; & ={\mathrm{FT}}_{2D}\left[\rho \left(x,y\right)\right] \end{array}$$

The backscattered data can be inferred by using 2-D IFT as

(7) $$\begin{array}{c} s\left(x,y,k\right)={\mathrm{IFT}}_{2D}\left[℘\left(k_{x},k_{y}\right)\frac{\exp(jk_{z}z_{0})}{k_{z}}\right] \end{array}$$

After dropping the constant components, it yields that

(8) $$\begin{array}{c} ℘\left(k_{x},k_{y}\right)=S\left(k_{x},k_{y},k\right)\exp\left(-jk_{z}z_{0}\right), \end{array}$$

where $S\left(k_{x},k_{y},k\right)={\mathrm{FT}}_{2D}\left[s\left(s,y,k\right)\right]$. To extract the complex reflectivity at a point on the Oxy plane, the 2-D IFT is applied to (8). By coherently summating all complex reflectivity at all points within the target plane, the 2-D image is constructed

(9) $$\begin{array}{c} \rho \left(x,y\right)=\int {\mathrm{IFT}}_{2D}\left[S\left(k_{x},k_{y},k\right)\exp\left(-jk_{z}z_{0}\right)\right]dk \end{array}$$

### 2.2. Data Collecting from 2-D Scanning System and RMA Involvement

**Figure 3:**
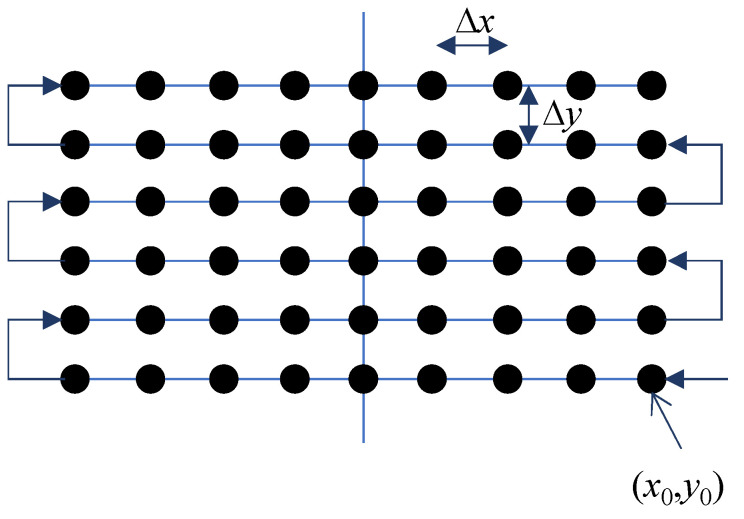
The measurement trajectory.

As mentioned in Section 2.1, the radar scans on the 2-D aperture plane. Let us denote the positions of the platform as $\left(x_{0},y_{0}\right)$ and $\left(x_{H_{N}},y_{V_{M}}\right)$ at the initial and final points on the aperture plane, respectively, where $H_{N}$ and $V_{M}$ denote the number of measurement points on each direction. The FMCW radar moves along the horizontal and vertical directions on the *x*-axis and *y*-axis in the zigzag trajectory, as shown in Figure 3. In this figure, the radar is moved with the horizontal and vertical steps of Δx and Δy, respectively. The measurement step must satisfy the Nyquist sampling which is dependent on the aperture size (Dx,Dy), target dimension ($Dt_{h}$ and $Dt_{W}$ are the height and the width of the target, respectively), and the distance between the antenna and the target ($z_{0}$)

(10) $$\begin{array}{c} \Delta x\leq \frac{\lambda \sqrt{{\left(Dx+Dt_{w}\right)}^{2}/4+z_{0}^{2}}}{2(Dx+Dt_{w})},\\ \Delta y\leq \frac{\lambda \sqrt{{\left(Dy+Dt_{h}\right)}^{2}/4+z_{0}^{2}}}{2(Dy+Dt_{h})}, \end{array}$$

where λ is the wavelength. To maintain the quality of the image, the spatial sampling intervals should be based on the worst cases of the Nyquist sampling where the $z_{0}\approx 0$, the Δx=Δy≤λ/4. However, to achieve high-quality imaging but with fewer measurements, these sampling intervals can be larger than the worst case of the Nyquist criterion or we can apply the CS-based method, which is presented in the next section. Since we are considering the 2-D imaging on the aperture plane, the range resolution (δz) is not so important. Instead, we take the cross-range resolutions (or azimuth resolutions) into account, which can be expressed as:

(11) $$\begin{array}{c} \delta x\approx \frac{\lambda }{2Dx},\;\delta y\approx \frac{\lambda }{2Dy}, \end{array}$$

where δx and δy correspond to the horizontal and vertical resolutions, respectively.

The 3-D data cube of the FMCW echo is accumulated after measuring all measurement points. Let us denote the received signal at the measurement point $\left(x_{i},y_{j}\right)$ as s(i,j), and each chirp contains $N_{s}$ samples, the collection of chirp signal data at all point having size $N_{s}\times V_{M}\times H_{N}$ can be formed as

(12) $$\begin{array}{c} S=\left[\begin{array}{cccc} s(H_{N},1) & s(H_{N},2) & \cdots & s(H_{N},V_{M})\\ \cdots & \cdots & \cdots & \cdots \\ s(1,2) & s(2,2) & \cdots & s(2,V_{M})\\ s(1,1) & s(1,2) & \cdots & s(1,V_{M}) \end{array}\right] \end{array}$$

In this study, we applied the FIR filter and window smoothing method to reduce interference from the environment. Besides that, we also apply an integral technique to improve the signal-to-noise ratio [36] as

(13) $$\begin{array}{c} s\left(x,y,k\right)=\int_{0}^{T}s\left(x,y,t\right)\exp\left(-j2\pi f_{b}t\right)dt \end{array}$$

where $f_{b}$ is the beat frequency of the IF signal at the corresponding distance $z_{0}$. Now, the RMA is applied by firstly transforming the data using range-FFT (fast Fourier transform) which can be expressed as F(S). After that, we perform the range focusing on the target to get the planar data at distance $z_{0}$, denoted as $M=F(S,k_{z_{0}})$ with the dimension of $V_{M}\times H_{N}$. Next, we define the matched filter as

(14) $$\begin{array}{c} \mathrm{MF}=\exp(-j2k\sqrt{x^{2}+y^{2}+{\left(z_{0}\right)}^{2}}). \end{array}$$

The image can be constructed as

(15) $$\begin{array}{c} \hat{M}={\mathrm{IFT}}_{2D}\left\{Stolt\to \left[{\mathrm{FT}}_{2D}\left(M⊗\mathrm{MF}\right)\right]\right\} \end{array}$$

where ⊗ denotes the element-wise multiplication operator for matrices. We adopt STOLT interpolation here into the image reconstruction to take the uniformly distributed data at the $k_{z}$ domain.

### 2.3. Compressed Sensing

In this section, we describe the participation of CS in the image scheme to improve the performance of the system. The involvement of CS contributes two main benefits to the imaging scheme. First, during the measurement process, the radar collects the echo signal by scanning on the 2-D aperture plane consisting of numerous measurement points which leads to a significantly long duration to accumulate all the signal data. With CS, the number of measurement points can be considerably decreased as the radar only emits and receives the signal at certain points as on the random measurement matrix. Secondly, CS helps to diminish the side-lobe effect caused by the non-uniform spacing of measurement points [31] which can bring about the ghost effect on the image result. The details of CS algorithms are presented in the next subsections.

#### 2.3.1. CS Theory

**Figure 4:**
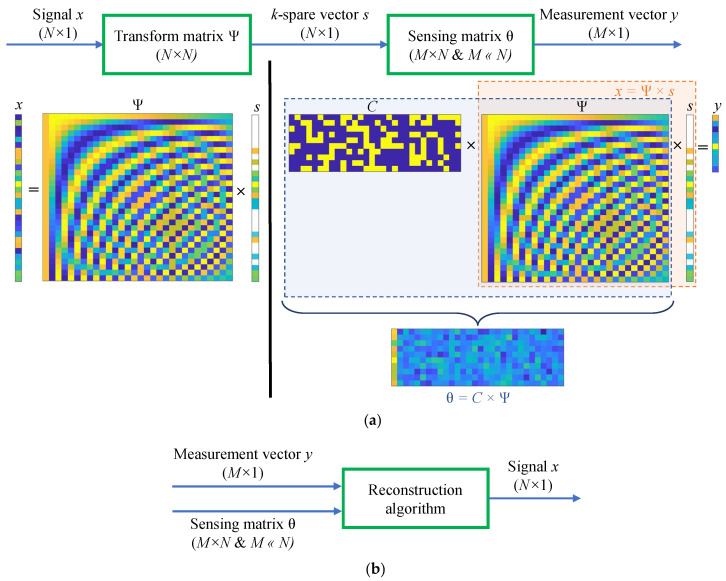
Compressed sensing processes: (**a**) signal compression and (**b**) signal reconstruction.

Signal acquisition is the main topic in signal processing. Sampling theorems provide the bridge between the continuous and the discrete-time worlds. The most famous theorem is often attributed to Shannon (but is usually called the Nyquist theorem) and states that the sampling rate must be twice the maximum frequency present in the signal to perfectly recover the signal. In 2004, David Dohono [21] proved that a signal can be reconstructed with fewer samples than the sampling theorem, which introduced the concept of compressive sampling. CS allows compressing signals with sparse or compressible representation while they are sampled. It initiates from the idea that it is not necessary to invest a lot of power into observing the entries of a sparse signal because most of them will be zero. The fundamental principle of the CS method can be described in Figure 4, which contains two phases, namely compress and recovery operations.

(1) A signal *x* with length N $\left(x\in R^{N\times 1}\right)$ needs to be reconstructed from *M* measurements such that M≪N.

(2) The signal is then made sparse through some transform matrix Ψ as:

(16) $$\begin{array}{c} x=\Psi s \end{array}$$

where *s* is the k−sparse coefficient vector of size N×1$\left(s\in R^{N\times 1}\right)$, and Ψ is the transform matrix whose size of N×N.

(3) With knowledge of the sparse vectors, it is possible to reconstruct signal *x* from (16). Thus, the goal of compressed sensing is to find the sparsest vector *s* that is consistent with the measurements vector. The measured signal vector is calculated as:

(17) $$\begin{array}{c} y=Cx=C\Psi s=\theta s \end{array}$$

where *y* is the measurement vector or the compressed signal of size M×1, θ=CΨ is the random sensing matrix of size MtimesN with $C\in R^{M\times N}$ being the measurement matrix. It is important to note that the measurement matrix *C* is not selected randomly, it must possess the RIP (restricted isometry property) condition, which means that it is guaranteed to hardly change the length of vector *x* as long as the vector *x* is at least k-sparse [37]. In [38], researchers show that the measurement matrix *C* adopting a random Gaussian matrix can probably satisfy the RIP condition. Another study [39] employs the random Bernoulli matrix as it extends the symmetric signs ensemble which also satisfies RIP with high probability.

(4) The reconstruction of the original signal can be seen as the process of solving y=θs. As only the sparsity *k* is given, this equation is an under-determined equation system. Hence, *s* then be recovered by finding the optimal $l_{0}$-norm to minimize the number of non-zero items in *s* which is a difficult problem. However, if we turn this optimization problem into an $l_{1}$-norm problem, it can be solved by using convex optimization [29]. The recovering process of *s* is as follows:

(18) $$\begin{array}{c} s^{*}=\arg\min{∥\theta s-y∥}_{2}<\epsilon \end{array}$$

where ${∥.∥}_{p}$ is the p−norm and ϵ is the residual.

#### 2.3.2. SAR Imaging with Compressed Sensing

For the application of CS, the sparse data are formed by reducing the dataset of reflected signals, which means that a smaller number of measurement points should be randomly taken in the aperture plane. In this paper, we selected the measurement points by applying the random Bernoulli matrix as the measurement matrix *C*. Let us express the data matrix with irregular data acquisition as $\tilde{S}$, it can be seen as a matrix in which random positions in S are set to zero. As most of the elements in $\tilde{S}$ are zeros, we can recover the data by using the $l_{1}-minimization$ method. Assuming the size of $\tilde{S}$ is $N_{s}\times {\tilde{V}}_{M}\times {\tilde{H}}_{N}$, where ${\tilde{V}}_{M}\ll V_{M}$ and ${\tilde{H}}_{N}\ll H_{N}$. We also assume that at the *i*th sample of all received signals at all measurement points, the received signal vector is expressed as $\tilde{S}\left(u,v,w=i\right)$. The signal vector $s^{*}$ at the *i*th sample can be restored by solving the minimization problem. In this study, three methods, namely ADMM, IRLS, and CD, are investigated to solve our problem. These methods can be expressed as below:

- The ADMM algorithm has been shown as a simple, effective, and fast convergent approach [40,41]. The optimization problem applied by the ADMM algorithm is given as
  (19) $$\begin{array}{c} s^{*}=\arg\min\left\{\frac{1}{2}{∥\theta s-y∥}_{2}+\lambda {∥z∥}_{1}\right\}\;\mathrm{subject}\;\mathrm{to}\;s=z \end{array}$$
  The regularization parameter λ (> 0) controls the balance between the data fidelity term $\frac{1}{2}{∥y-\theta s∥}_{2}$ and the regularization term ${∥z∥}_{1}$. The update equations of ADMM over iteration for (19) are given by
  $$\begin{array}{cc} s^{\left[k+1\right]} & =\arg\min\left\{\frac{1}{2}{∥\theta s-y∥}_{2}+\frac{\eta }{2}{∥s-z^{\left[k\right]}+w^{\left[k\right]}∥}_{2}\right\} \end{array}$$
  (20) $$\begin{array}{cc} \; & ={\left(\theta^{T}\theta +\eta I\right)}^{-1}\left(\theta^{T}+\eta \left(z^{\left[k\right]}+w^{\left[k\right]}\right)\right)\\ z^{\left[k+1\right]} & =\arg\min\left\{{\lambda ∥z∥}_{1}+\frac{\eta }{2}{∥s^{\left[k+1\right]}-z+w^{\left[k\right]}∥}_{2}\right\} \end{array}$$
  (21) $$\begin{array}{cc} \; & ={\mathrm{prox}}_{\left(\frac{\lambda }{\eta }f\right)}\left(s^{\left[k+1\right]}+w^{\left[k\right]}\right) \end{array}$$
  (22) $$\begin{array}{cc} w^{\left[k+1\right]} & =w^{\left[k\right]}+s^{\left[k+1\right]}-z^{\left[k+1\right]} \end{array}$$
  where k(=0,1,2,⋯) is the iteration index in the algorithm, ${\left(.\right)}^{T}$ is the transpose of the matrix, I is the identify matrix, and η(>0) is the parameter. The initial values of $w^{\left[0\right]}$ and $z^{\left[0\right]}$ are 0.
- In the IRLS method, the weight least square in each iteration is used to infer the next estimate with the weights derived from the last iteration. The optimization problem in each iteration is as follows: ${s^{*}}^{n+1}=\arg\min s^{T}W^{n}s\;\mathrm{subject}\;\mathrm{to}\;y=\theta s$, n≥1, where *W* is the diagonal weight matrix. At the *k*th iteration, this matrix is computed from the solution of the current iteration $s^{k}$, $W_{i}^{k}={\left|s_{i}^{k}\right|}^{-1}$, 1≤i≤N. The closed form solution for $s^{k+1}$ can be inferred as $s^{k+1}={\left(W^{k}\right)}^{-1}\theta^{T}\left(\theta {\left(W^{k}\right)}^{-1}\theta^{T}\right)y$.
- The coordinate descent method can be applied in multi-variable minimization by solving a sequence of scalar minimization sub-problems. By minimizing each sub-problem along a selected coordinate while all other coordinates are fixed, the estimate of the solution is improved [42].

## 3. Experiment Setup and Results

In this chapter, the measurement system for FMCW SAR imaging is presented including the descriptions of all the hardware components and their integrations into the system to collect echo data from the target. After that, the system configuration of the radar and the experiment setups for collecting data are also introduced, followed by their experiment results.

**Figure 5:**
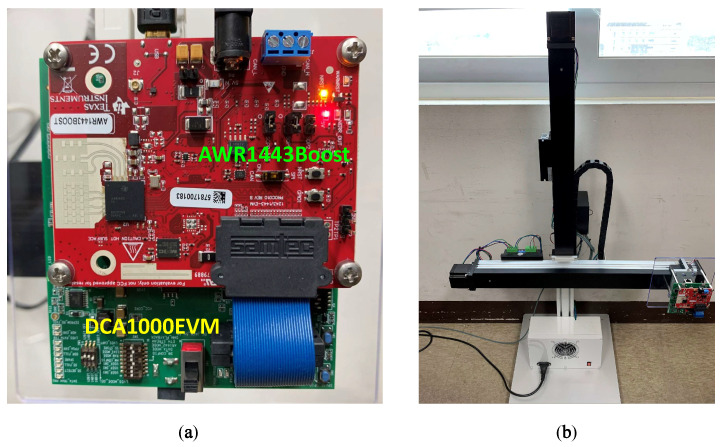
Measurement system (**a**) the TI AWR1440Boost with DCA1000 data card and (**b**) the 2-D motion platform.

The mmWave radar in our system is the Texas Instrument AWR1443Boost, which is an evaluation module based on a single-chip AWR1443 mmWave sensor. There are three transmit antennas and four receive ones integrated into the radar. It can emit an FMCW signal with a 4 GHz available bandwidth in the frequency range from 76 GHz to 81 GHz [43]. Besides that, it is also configured to be interfaced with the DCA1000 evaluation module, as shown in Figure 5a. This DCA1000 module is a real-time data capture board for interfacing with Texas Instrument’s mmWave sensors. The raw ADC data recorded by AWR1443Boost are then captured by the DCA1000 module and streamed to the host PC over Ethernet as packetized data. The designed moving platform in our system has two orthogonal linear actuators, as shown in Figure 5b.

### 3.1. Experiment Setup

**Figure 6:**
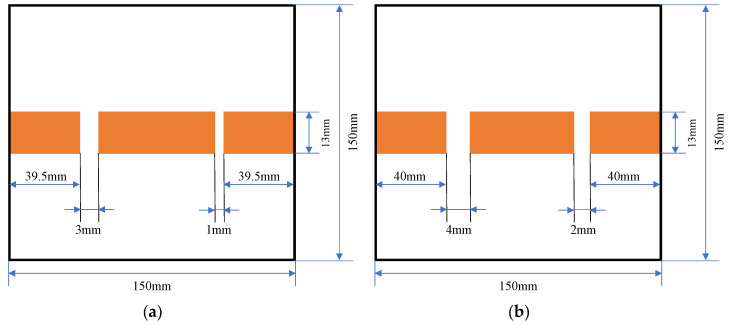
Reinforced concrete sample structures consisting of: (**a**) 1 mm and 3 mm cracks (sample A) and (**b**) 2 mm and 4 mm cracks (sample B).

In this study, we take into consideration the feasibility of integrating the system into the NDT applications. To archive that, we conduct an NDT experiment by taking the involvement of the reinforced concrete as the MUT (material under test). This experiment strives to locate the cracks in the steel rebar inside the concrete samples. We conduct the measurements on three reinforced concrete samples, one sample has two gaps (cracks) on the rebar with dimensions of 1 mm and 3 mm, and another sample contains two cracks of 2 mm and 4 mm on its rebar (we named them sample A and sample B). To make the concrete sample easier to produce, every crack is filled with a silicon layer whose low reflection ability is compared to the steel rebars. Each rebar is 150 mm long and its diameter is 13 mm. The distance between the surface of the concrete to its rebars is around 2 mm. Figure 6 shows the structure of the reinforced concrete samples with a crack on their rebars.

**Figure 7:**
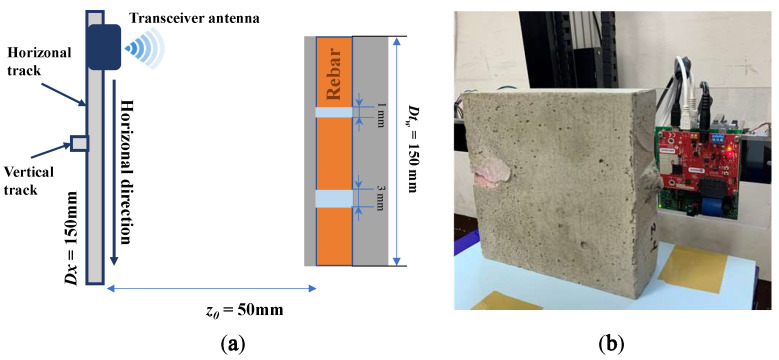
Non-destructive testing measurement setup: (**a**) the top view and (**b**) the real setup.

Based on these characteristics of the concrete, we consider $Dt_{w}=150$ mm and $Dt_{h}=13$ mm. Therefore, to make sure the coverage of the scanning region on the target, the dimension of the scanning plane is defined as Dx=150 mm and Dy=40 mm. The movement steps in both horizontal and vertical directions are Δx=1 mm and Δy=2 mm, respectively, which results in 3000 measurement points including 150 points in the horizontal direction and 20 points in the vertical one. Because of the concrete layer covering the rebar, we decide to utilize the maximum antenna gain, which is set to 48 dB. To validate the different azimuth resolutions on the image which play a crucial role in detecting hidden cracks, we carry on the experiments on two different values of $z_{0}$, 100 mm and 50 mm. The experiment setup for detecting cracks in the rebar of concrete is shown in Figure 7.

### 3.2. Experiment Results

**Figure 8:**
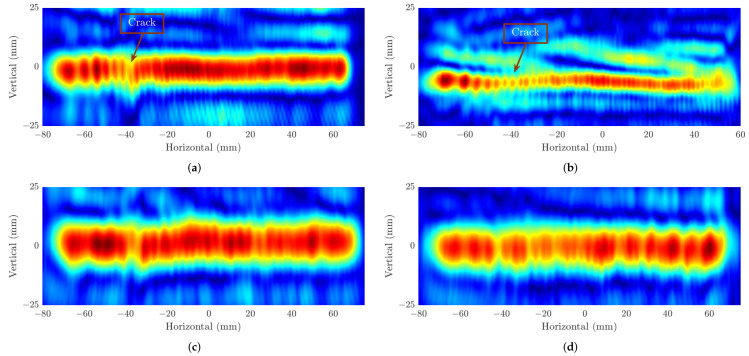
SAR image results at distance $z_{0}=50$ mm of (**a**) sample A, (**b**) sample B, and at $z_{0}=100$ mm of (**c**) sample A and (**d**) sample B.

According to the theory of the azimuth resolution in (11), as the range between the radar to the target $\left(z_{0}\right)$ increases, the resolution is decreased correspondingly. Our image results in Figure 8 confirm the negative correlation between these two factors in the SAR algorithm. These range values of $z_{0}$ at 50 mm and 100 mm and experiments configuration in Section 2.2 theoretically lead to the theoretical resolutions of (δx≈ 1 mm, δy≈ 2.4 mm) and (δx≈ 1.3 mm, δy≈ 4.9 mm), respectively. For all the experiments, the same dimensions of the 3-D data cubes are captured, 512×20×150 corresponding to $N_{s}\times V_{M}\times H_{N}$. Figure 8a,b illustrates the image results of the two first experiments on concrete samples where the distance from the FMCW radar to the steel rebar $\left(z_{0}\right)$ is 50 mm. It can be seen that the proposed imaging scheme can determine the positions of the rebars hidden within the concretes. In terms of crack detection application, these experiments show inefficient abilities since it is only able to determine the position of 3 mm and 4 mm gaps, while the small cracks whose sizes of 1 mm and 2 mm do not appear in these figures. Even though the theoretical horizontal resolution for this experiment is around 1 mm, the ghost effect on the image that comes from multi-static measurements makes it so the tiny gaps are not located. In addition, another reason for this result is that the reflection of the silicon layers filled in the gaps can be shown as parts of the rebars on the SAR images. Although the weak echo is reflected from those layers, the gaps cannot be distinguished. The SAR image results of the experiments carried out on the same concrete samples but different from the $z_{0}=100$ mm are illustrated in Figure 8c,d. As shown in these images, although there are weaker reflections at the positions of the cracks than in the other parts of the rebars, it is difficult to conclude whether these positions contain the cracks or not without reference to the real structure of the concrete. There are two main factors that can explain this result. The first one is the lower theoretical resolution from the experiment setup, while the second one is the echo from the silicon layer within the gaps as in the previous experiment on concrete samples.

**Figure 9:**
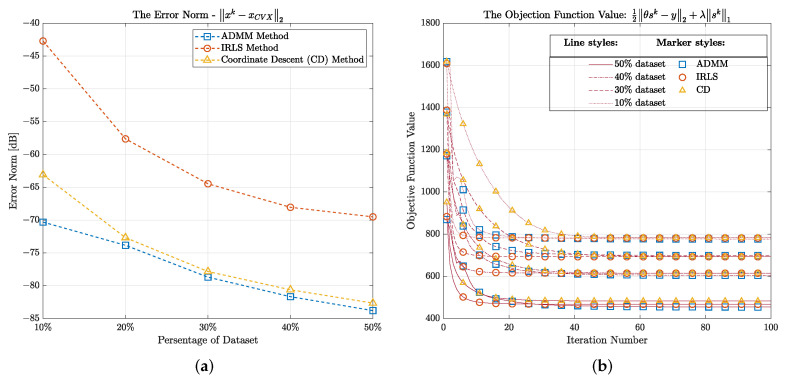
(**a**) The reconstruction error and (**b**) the convergence of three CS solvers.

Next, we validate the integration of CS algorithms into the imaging scheme. We first take smaller parts of the dataset from the original one and then try to reconstruct the SAR image from the recovered data by adopting CS. We apply the Bernoulli random matrix (C) to take the amount of 10%, 20%, 30%, 40%, and 50% from the original data. We use the discrete cosine transform (DCT) as the transform matrix (Ψ). After that, the three aforementioned CS-based solvers are used, which involved restoring the data from these partitions. The performance of these three solvers is depicted in Figure 9, including the evaluation of their reconstruction errors and their convergence. To validate the reconstruction error, for each CS solver method, we infer the error norm between the reconstructed data from them and the one from the convex solver at all five amounts of the dataset, which is expressed as $Err=∥x^{k}-x_{CVX}{∥}_{2}$, where *k* (= 1, 2, 3) represents for the order of three solvers (ADMM, IRLS, and CD). It can be seen in Figure 9a that, for all solvers, as we increment the amount of data in of dataset, the reconstructed error is reduced. More specifically, ADMM brings about the lowest error from the recovered data at all parts which means that it performs better than its counterparts in our problem. On the other hand, Figure 9b reveals the convergence of this solver after 100 iterations. It again confirms the negative correlation between the amount of data and the objective function value in terms of finding the minimum solution as, the higher percentage of the dataset, the lower value of the objective function. Overall, CD takes more iterations than other methods to converge which leads to a longer duration to recover the data, while IRLS takes the least time in the same task.

**Figure 10:**
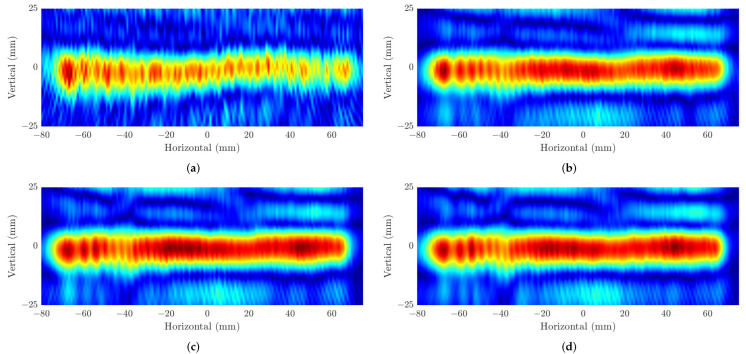
SAR images from the data recovered by the ADMM method employing (**a**) 10% data, (**b**) 30% data, (**c**) 40% data, and (**d**) 50% data.

Finally, the formed SAR images from the recovered data by adopting ADMM solvers on four parts of data including 10%, 30%, 40%, and 50% from the original one are shown in Figure 10. Here, we reveal the images of the rebar consisting of cracks of 1 mm and 3 mm when the distance $z_{0}=50$ mm. In Figure 10a, due to the high reconstructed error from the small amount of data, it illustrates the distortion on the shape of the rebar resulting in the undetectable crack positions on it. On the contrary, the SAR images formed by using 30% to 50% of the data (Figure 10b,d) present higher quality with less distorted segments, which can help to determine the location of the defect on the MUT. Nevertheless, the low contrast in Figure 10b, where only 30% of data is employed, still makes the defect slightly difficult to be recognized compared to Figure 10c,d, where the 3 mm crack can be shown as the same as the original in Figure 8a.

## 4. Conclusions

In this paper, we describe the design, development, and evaluation of the SAR imaging system adopting the mmWave signal from FMCW radar. Our scheme demonstrates its ability to detect the defect in the object by locating its position and constructing its 2-D surface shape, which can support finding and determining the failures when that object is invisible to human eyes. Specifically, we adopted the imaging scheme to the NDT application for the reinforced concrete and although it failed to detect the small cracks in its rebar, it works well in cases of larger cracks. Additionally, this proposed system can take part in various see-through-wall applications from detecting concealed weapons, structure health monitoring, and material assessment during manufacturing to finding and saving living objects under the wreckage after a disaster. To archive the 2-D imaging of the object, the RMA method was investigated along with the digital signal processing techniques applied to remove the undesired signal and improve the image quality. Besides that, the involvement of the CS algorithm in the imaging system helps to enhance the performance of the system as it significantly reduces the requirements for spatial data acquisition and the side-lobe effect from the non-uniform spacing of measurement points. Three CS-based reconstruction algorithms, namely ADMM, IRLS, and CD, are presented to reconstruct the data with spare samples. The results confirm that the scheme can form the image even with a sparsity rate of 30%, while the ADMM algorithm shows better performance. In the future, the methods to improve the azimuth resolution of the system need to be researched for identifying tiny defects by joining the AI algorithms and placing virtual antenna elements on wider non-uniform samples.

## Data Availability

Not applicable.
